# Supplementary material for: Stacking Model‐Based Classifiers for Dealing With Multiple Sets of Noisy Labels
Source: Biom J. 2025 Mar 12;67(2):e70042. doi: 10.1002/bimj.70042 (PMC11898607; doi:10.1002/bimj.70042)
Supplement: Supplementary file 1 — Supporting Information [file BIMJ-67-e70042-s001.zip › density-based-ensemble-model-main/competitors_real_data/netlab-master/netlab-master/help/gpinit.htm]

Netlab Reference Manual gpinit


# gpinit

## Purpose

Initialise Gaussian Process model.

## Synopsis

```
net = gpinit(net, trin, trtargets, prior)
net = gpinit(net, trin, trtargets, prior)
```

## Description

`net = gpinit(net, trin, trtargets)` takes a Gaussian Process data structure `net`
together
with a matrix `trin` of training input vectors and a matrix `trtargets` of
training target
vectors, and stores them in `net`. These datasets are required if
the corresponding inverse covariance matrix is not supplied to `gpfwd`.
This is important if the data structure is saved and then reloaded before
calling `gpfwd`.
Each row
of `trin` corresponds to one input vector and each row of `trtargets`
corresponds to one target vector.

`net = gpinit(net, trin, trtargets, prior)` additionally initialises the
parameters in `net` from the `prior` data structure which contains the
mean and variance of the Gaussian distribution which is sampled from.

## Example

Suppose that a Gaussian Process model is created and trained with input data `x`
and targets `t`:

```
net = gp(2, 'sqexp');
net = gpinit(net, x, t);
% Train the network
save 'gp.net' net;
```

Another Matlab program can now read in the network and make predictions on a data set
`testin`:

```
load 'gp.net';
pred = gpfwd(net, testin);
```

## See Also

`gp`, `gpfwd`

---

**Pages:**
Index


---

Copyright (c) Ian T Nabney (1996-9)
